# Overexpression of CD6 and PD-1 Identifies Dysfunctional CD8^+^ T-Cells During Chronic SIV Infection of Rhesus Macaques

**DOI:** 10.3389/fimmu.2019.03005

**Published:** 2020-01-08

**Authors:** Gospel Enyindah-Asonye, Anthony Nwankwo, Mohammad Arif Rahman, Ruth Hunegnaw, Christopher Hogge, Sabrina Helmold Hait, Eun-Ju Ko, Tanya Hoang, Marjorie Robert-Guroff

**Affiliations:** Vaccine Branch, National Cancer Institute, National Institutes of Health, Bethesda, MD, United States

**Keywords:** rhesus macaque, simian immunodeficiency virus, T-cell exhaustion, CD6, PD-1

## Abstract

Effective CD8^+^ T-cell responses play an important role in determining the course of SIV/HIV viral infection. Here we identified a unique population of dysfunctional CD8^+^ T-cells in lymphoid tissues and bronchoalveolar lavage (BAL) of rhesus macaques with chronic SIV infection characterized by co-expression of CD6 and PD-1. The frequency of CD6 and PD-1 co-expressing CD8^+^ T-cells was significantly increased in lymphoid tissues and BAL during chronic SIV infection compared to pre-infection levels. These CD6^+^PD-1^+^CD8^+^ T-cells displayed impaired proliferation, cytokine secretion and cytotoxicity compared to their CD6^−^PD-1^+^CD8^+^ T cell counterparts. The frequency of CD8^+^PD-1^+^ and CD8^+^CD6^−^PD-1^+^ T-cells in the lymph node and bone marrow did not correlate with SIV viral load, whereas the frequency of CD8^+^CD6^+^PD-1^+^ T-cells positively correlated with SIV viral load in these tissues highlighting the contribution of CD6 to disease progression. CD6^+^PD-1^+^CD8^+^ T-cells expressed elevated levels of SHP2 phosphatase compared to CD6^−^PD-1^+^CD8^+^ T-cells providing a potential mechanism by which CD6 may induce T-cell dysfunction during chronic SIV infection. Combined targeting of CD6 and PD-1 effectively revived the CD8^+^ T-cell proliferative response *in vitro* suggesting a strategy for potential therapeutic benefit.

## Introduction

CD8^+^ T-cells play a central role in defense against viral infections (1–3). Their ability to curtail viral replication is compromised when they become functionally impaired or exhausted leading to diminished proliferation, cytokine secretion and ability to lyse virus-infected cells (4–6). Overexpression of multiple inhibitory receptors resulting in attenuated T-cell receptor (TCR) signaling and immune responses has been implicated in CD8^+^ T-cell exhaustion during chronic infections (6, 7). The lack of optimal CD8^+^ T-cell responses is a major cause of viral disease persistence and progression.

PD-1 (program death 1), a hallmark marker for dysfunctional CD8^+^ T-cells in viral infections such as HCV (8), HIV (9), and SIV (4), is a cell surface receptor protein expressed on T-cells, B-cells and myeloid derived cells (10). It functions primarily to attenuate immune responses (11). PD-1 expression on activated CD8^+^ T-cells is normally transient and helps prevent hyperactivation and autoimmunity (12), but chronic antigen stimulation results in epigenetic changes and prolonged and sustained high PD-1 levels on CD8^+^ T-cells (12). *In vivo* blockade of PD-1 in rhesus macaques has been shown to be therapeutically beneficial (13, 14). However, several studies indicate that blockade of the PD-1 pathway alone fails to completely restore T-cell function, suggesting involvement of other inhibitory pathways in CD8^+^ T-cell dysfunction (4, 13–15).

CD6 is a transmembrane receptor primarily expressed on T-cells (16) and B1a cells (17). Its influence on T-cells has been controversial due to contradictory *in vitro* findings obtained using various CD6 targeting monoclonal antibodies (mAbs) suggesting either a co-stimulatory or inhibitory role in T-cell activation (18–21). Recent studies utilizing CD6-deficient mice suggested that CD6 is a co-inhibitory molecule that inhibits T-cell responses (22, 23). Additionally, over-expression of CD6 on human PBMC restrained T-cell activation, cytokine release and proliferation, indicating that CD6 attenuates T-cell responses (24). The tyrosine phosphatase, SHP2, was reported to interact with CD6, providing the first biochemical evidence of a mechanism by which CD6 could inhibit T-cell responses (19). SHP2 is an effector molecule downstream of the PD-1 inhibitory signaling pathway in T-cells suggesting that CD6 may synergize with PD-1 to inhibit T-cell responses (25). CD6 has been implicated in the pathogenesis of several autoimmune diseases and has become a therapeutic target (26, 27). Recently a mAb targeting CD6 was approved for the treatment of chronic plaque psoriasis (28).

Whether the combined effects of CD6 and PD-1 co-expression on CD8^+^ T-cells contribute to SIV disease progression is not known. Here, we report that CD6 and PD-1 overexpression on CD8^+^ T-cells identifies a population that arises in lymphoid tissues during chronic SIV infection, displays impaired anti-viral responses, and is associated with SIV disease progression. Our data point to CD6 as a potential novel therapeutic target to revive dysfunctional CD8^+^ T-cells during chronic infection.

## Materials and Methods

### Study Animals

Rhesus macaques were maintained at Advanced Bioscience Laboratories, Inc. (Rockville, MD) and at the National Cancer Institute animal facility (Bethesda, MD) under the guidelines of the Association for the Assessment and Accreditation of Laboratory Animal Care and according to the recommendations of the *Guide for the Care and Use of Laboratory Animals*. Protocols and procedures were approved by the Institutional Animal Care and Use Committee of each facility. Bone marrow, inguinal lymph node (LN) and blood samples were collected in this cross-sectional study from macaques in Vaccine Study I (Table 1) prior to immunization, during the course of vaccination, and following SIV infection at acute (2 weeks post-infection; 2 wpi) and chronic (medians of 40–42 wpi) time points. Spleen samples were collected from chronically infected macaques only at necropsy. In addition, bronchoalveolar lavage (BAL) samples were longitudinally obtained prior to vaccination and at acute and chronic time points from 7 macaques in Vaccine Study II (Table 1). Plasma viral loads were assayed by nucleic acid sequence-based amplification (29).

**Table 1:** Summary of rhesus macaques used in this study.

**Study**	**Vaccine arm**	**Number macaques**	**Sex**		**MHC haplotype**		**Weeks infected^a^**	**Chronic viral load**	**CD4 count^b^**
			**F**	**M**	Mamu A*01	Mamu B*17	**(Median)**	**(Median; wks 12–40)**	**(Median absolute counts)**
I	ALVAC/Env	19	12	7	2	2	42	1.12 × 10^6^	475
I	DNA and Env	18	9	9	2	1	42	1.62 × 10^6^	470
I	Adjuvant controls	5	2	3	1	1	40	2.10 × 10^6^	314
II	Vaccine + gel	3	3	0	0	0	40	2.87 × 10^4^	377
II	Vaccine + microbicide	3	3	0	2	0	40	3.50 × 10^3^	400
II	Adjuvant controls + gel	1	1	0	0	0	41	1.27 × 10^3^	410

*Blood and tissue samples were obtained from macaques prior to, over the course of vaccination, and following SIV challenge as indicated in the text. In Vaccine Study I (Musich et al., manuscript in preparation)^1^ macaques were primed mucosally with replication-competent Adenovirus 5 host range mutant (Ad5hr) SIV recombinants encoding Env and Gag and boosted systemically with either ALVAC SIVgag,pro,env plus SIV gp120 in alum or with electroporated SIV DNA encoding Env, Gag, and rhesus IL-12 followed immediately with gp120 protein in alum. Control macaques received empty Ad5hr vector plus alum only. The macaques were challenged intrarectally with repeated low doses of SIV_mac251_. In vaccine Study II (Helmold Hait et al., manuscript submitted for publication)^2^ macaques were primed mucosally with Ad5hr-SIV recombinants encoding Env/Rev, Gag and Nef and were boosted with gp120 in alum adjuvant. Controls received Ad5hr empty vector and alum. The macaques were challenged intravaginally with repeated low doses of SIV_mac251_ with or without the presence of microbicide formulated in gel. The microbicide was shown to affect SIV acquisition but not viral loads post-infection*.

a *Indicates number of weeks macaques were infected prior to euthanasia*.

b *CD4 counts were obtained close to week 40 for study I and at 20 wpi for study II*.

### Sample Collection and Preparation

Spleen and inguinal lymph node (LN) biopsies were dissected and passed through a 40 μm cell strainer after lysis of red blood cells (RBCs). The cells were washed and resuspended in R10 media (RPMI 1640 containing 10% FBS, 2 mM L-glutamine, 1% nonessential amino acids, 1% sodium pyruvate, and antibiotics) (30–32). Bone marrow cells were isolated by density gradient centrifugation on Ficoll. Following lysis of RBCs, they were stored frozen in FBS-10% DMSO. BAL samples were obtained as previously described (33). The cells were pelleted, washed in cold PBS, and stored frozen in FBS-10% DMSO.

### Flow Cytometric Acquisition

Thawed cells were stained on ice for 30 min in the dark using manufacturers' suggested mAb concentrations, washed with PBS and resuspended in FACS buffer. 500,000 singlet events were acquired on a SORP LSR II (BD Biosciences) and analyzed using FlowJo software (FlowJo, Ashland, OR). Gating was established using a combination of isotype and fluorescence-minus-one controls.

### Antibodies

The following mAbs were used: anti-CD6 (MT-605), anti-CD4 (L200), anti-CD8 (RPA-T8), anti-CD3 (SP34.2), anti-LAG-3 (T47-530), anti-IFN-γ (B27), anti-granzyme B (GB11), and anti-CD45 (D058-1283) (all from BD Bioscience, San Jose, CA); anti-perforin (MABTECH, Cincinnati, OH); anti-PD-1 (EH12.2H7), anti-IL-2 (MQ1-17H12) and anti-TNF-α (MAB11) (all from Biolegend, San Diego, CA); anti-CD206 (19.2) (eBioscience, San Diego, CA); polyclonal rabbit anti-SHP2 (Lifespan Biosciences, Seattle, WA); anti-CD43 (4-29-5-10-21), rabbit IgG isotype control and goat anti-rabbit alexa-488 (Invitrogen, Carlsbad, CA); Control immunoglobulin (mIgG1) was obtained from Sigma-Aldrich (St. Louis, MO). Purified anti-human/monkey TNF-α mAb (MT21A8), biotinylated anti-human/monkey TNF-α mAb (MT15B15), streptavidin-HRP and human TNF-α standard recombinant were obtained from Mabtech (Cincinnati, OH). CM9 (Gag, CTPYDINQM; residues 181–189 FITC) dextramer was obtained from Immudex (Fairfax, VA).

### Flow Cytometric Detection of IL-2, TNF-α, IFN-γ, Perforin, Granzyme B, and SHP2

Splenocytes from chronically SIV-infected macaques were cultured in R10 in the presence of Golgistop (1 μl; BD) containing monensin for 4 h prior to surface staining with anti-CD3, anti-CD8, anti-CD6, and anti-PD-1. The cells were fixed and permeabilized using intracellular fixation and permeabilization buffer (eBioscience) and stained with anti-IL-2, IFN-γ, Perforin, Granzyme B, and anti-TNF-α. Isotype-matched mAbs served as negative controls.

For SHP2 detection, following surface staining and fixation and permeabilization as above, cells were stained with anti-SHP2, washed and stained with goat anti-rabbit IgG conjugated to alexa-488. After further washing the cells were analyzed by flow cytometry. Rabbit IgG isotype served as negative control.

For SIV-specific CD8^+^ T cell staining, spleen and LN cells from chronically SIV-infected Mamu A^*^01-positive macaques were incubated with 10 μl CM9 (Gag, CTPYDINQM; residues 181–189 FITC) dextramer in the dark at room temperature for 10 min. Following incubation, the cells were also stained with anti-CD8, anti-PD1, and anti-CD6 antibodies in the dark at 4°C for 20 min. After this final incubation step, the cells were washed and analyzed by flow cytometry.

### CFSE Proliferation Assay

Splenocytes from chronically infected animals were labeled with CFSE (carboxyfluorescein diacetate succinimidyl ester; Sigma-Aldrich, St. Louis, MO) and stimulated *in vitro* with anti-monkey CD3 (5 μg/mL; Mabtech, Cincinnati, OH) for 3 days. Proliferation was determined by loss of CFSE in CD8^+^CD6^+^PD-1^+^ and CD8^+^CD6^−^PD-1^+^ cells by flow cytometry.

### Cell Sorting

Splenocytes from chronically infected animals were stained with anti-CD4, anti-CD6, anti-CD8, and anti-PD-1. Blue Live/Dead viability dye was used to exclude dead cells. After washing, cells were passed through a 40 mm cell strainer and 3 populations were sorted on an Astrios EQ flow cytometer: CD8^+^PD-1^+^CD6^+^, CD8^+^ PD-1^+^CD6^−^, and CD4^+^with purity of 85%.

### Killing Assay

CD8^+^ T-cell cytotoxic activity was assayed as previously described (30). Sorted autologous CD4^+^ T-cells pulsed with or without SIV_mac239_ Gag pooled peptides (complete set of 15-mers overlapping by 11 amino acids; NIH AIDS Reagent Program) were used as targets and sorted CD8^+^PD-1^+^CD6^+^ or CD8^+^ PD-1^+^CD6^−^ cells were used as effectors. Specific killing was defined as percentage killing of peptide-pulsed targets minus percentage killing of targets without peptide pulsing.

### *In vitro* Blocking Experiment

Spleen cells from chronically infected animals were CFSE labeled and stimulated *in vitro* with 5 μg/mL of anti-monkey CD3 for 5 days in the presence of 20 μg/ml of anti-CD6 (clone UMCD6), anti-PD-1 (clone EH2.2H7), anti-CD6 plus anti-PD-1, or control mouse IgG1. Following stimulation, proliferation was analyzed by loss of CFSE in CD8^+^ T cells by flow cytometry.

### Statistical Analysis

For multiple group analyses, we performed the Kruskal-Wallis test and Dunn's multiple comparison test. For two group comparisons, we performed the nonparametric Mann-Whitney test. Correlation analyses were assessed using the nonparametric Spearman test. All tests were two-tailed and done at the 0.05 alpha level. GraphPad Prism was used for statistical analysis.

## Results

### Co-expression of CD6 and PD-1^+^ CD8^+^ T-Cells During Chronic SIV Infection

**Figure 1:**
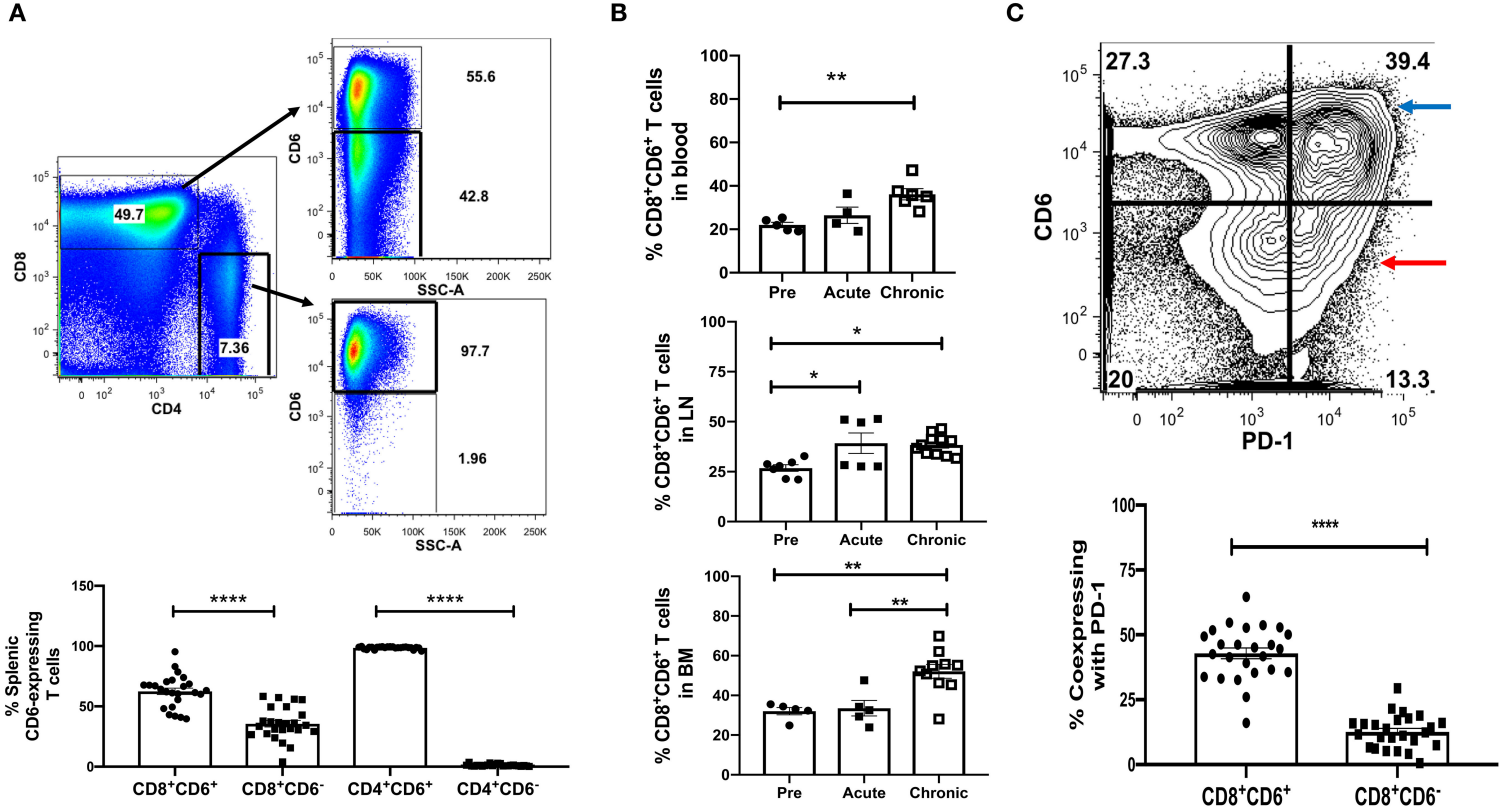
Characterization of CD6 and PD-1 expression on CD8^+^T-cells in different tissue compartments of chronically SIV-infected macaques. **(A)** Flow cytometry gating strategy for CD6 expression on CD8^+^ and CD4^+^ T-cells in the spleen during chronic infection (top panels) and frequencies of CD8^+^ and CD4^+^ T-cells expressing CD6 (bottom panel). Data are from 27 macaques. **(B)** The frequency of CD8^+^CD6^+^ T-cells in the blood (top row), LN (middle row), and bone marrow (bottom row) at different time points following SIV infection. Data for the blood are from 4 to 6 macaques, data for the LN are from 7 to 11 macaques, data for the bone marrow are from 5 to 10 macaques. **(C)** Representative staining for CD6 and PD-1 co-expression on splenic CD8^+^ T-cells during chronic SIV infection (top panels) and frequencies of CD8^+^CD6^+^ and CD8^+^CD6^−^ cells co-expressing PD-1 (bottom panel). Red arrow indicates CD8^+^CD6^+^PD-1^+^ cells, whereas blue arrow indicates CD8^+^CD6^−^PD-1^+^ cells. Data are from 25 macaques. Data are shown as means ± SEM. For statistical analysis, nonparametric Mann-Whitney tests were performed *p < 0.05, **p < 0.01, ****p < 0.0001.

We first examined CD6 expression during chronic SIV infection on CD8^+^ and CD4^+^ T-cells in the spleen. CD8^+^ T-cells exhibited two subsets; the mean frequency of CD6^+^CD8^+^ T-cells (62.5%) was 1.8-fold higher than that of CD6^−^CD8^+^ T-cells (35.6%) (Figure 1A). The majority (98%) of CD4^+^ T-cells were CD6^+^ (Figure 1A). We next examined CD8^+^CD6^+^ T-cells in lymphoid and non-lymphoid tissues of uninfected, acutely-infected, or chronically-infected macaques. The frequencies of CD8^+^CD6^+^ T-cells in the blood, LN and bone marrow were significantly expanded in the chronic group compared to the pre-infection group (Figure 1B). Splenocytes were not included as naïve splenocytes were unavailable. To determine whether the expansion of CD8^+^CD6^+^ T cells was an outcome of chronic infection and not vaccination, we assessed the dynamics of CD8^+^CD6^+^ T cells throughout vaccination before infection. We found that the frequencies of CD8^+^CD6^+^ T cells in the blood and lymph node were not altered (Supplementary Figure 1A). We also assessed the expression level of CD6 on CD8^+^ T cells throughout vaccination. The expression levels of CD6 were also not altered (Supplementary Figure 1B). These data suggested that vaccination had no significant influence on CD6 over-expression on CD8 T cells and that expansion of CD8^+^CD6^+^ T cells resulted from chronic infection. PD-1 expression on CD8^+^ T-cells is linked to chronic SIV disease progression. Therefore, we assessed whether CD6 and PD-1 are expressed by distinct or overlapping populations of CD8^+^ T-cells during chronic infection. The frequency of PD-1 co-expression on CD6^+^CD8^+^ T-cells (42.9%) was significantly greater compared to CD6^−^CD8^+^ T-cells (12.7%) (Figure 1C). We also detected another CD8^+^ T-cell population expressing only CD6 but not PD-1 (CD6^+^PD1^−^) during chronic infection.

### Overexpression of CD6 and PD-1 Is Associated With CD8^+^ T-Cell Dysfunction During Chronic SIV Infection

**Figure 2:**
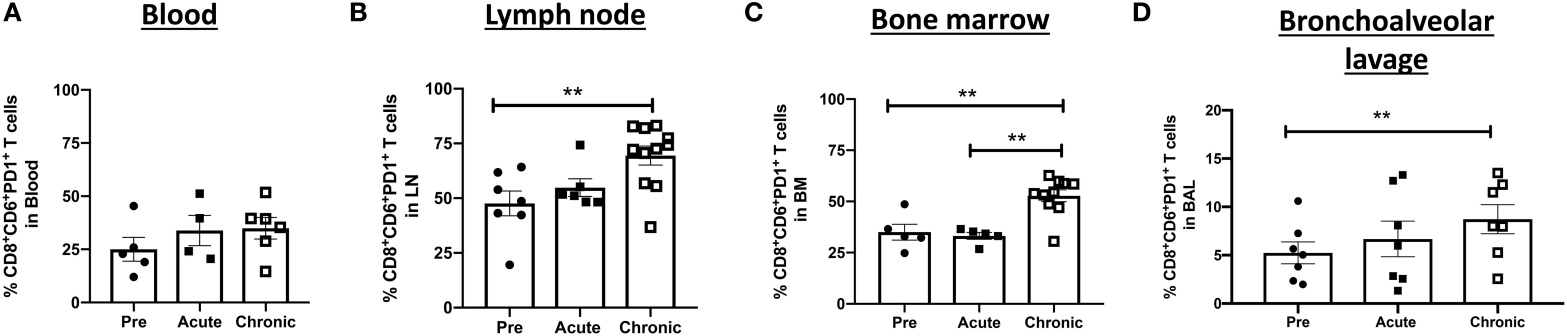
Dynamics of CD6 and PD-1 expressing CD8^+^ T-cells over the course of SIV-infection. The frequency of CD8^+^CD6^+^PD-1^+^ T-cells in the **(A)** blood, **(B)** inguinal LN, **(C)** bone marrow, and **(D)** BAL at different time points following SIV infection. Data for the blood are from 4 to 6 macaques, data for the LN are from 7 to 11 macaques, data for the bone marrow are from 5 to 10 macaques, data for BAL are from 7 macaques. For statistical analysis, Kruskal-Wallis with Dunn's multiple comparison tests were performed. **p < 0.01.

We next assessed the dynamics of CD8^+^CD6^+^PD1^+^ T-cells over the course of SIV infection in different tissue compartments. We hypothesized that these cells would be expanded during chronic SIV infection if CD6 and PD-1 co-expression were associated with T-cell dysfunction. The frequencies of CD8^+^ T-cells co-expressing CD6 and PD-1 were not significantly different between acutely-infected and pre-infection groups in the blood, LN, bone marrow, and BAL (Figures 2A–D). However, as anticipated, the frequency of CD8^+^ T-cells co-expressing CD6 and PD-1 in the LN, bone marrow and BAL was significantly expanded in the chronic group compared to the pre-infection group (Figures 2B–D).

**Figure 3:**
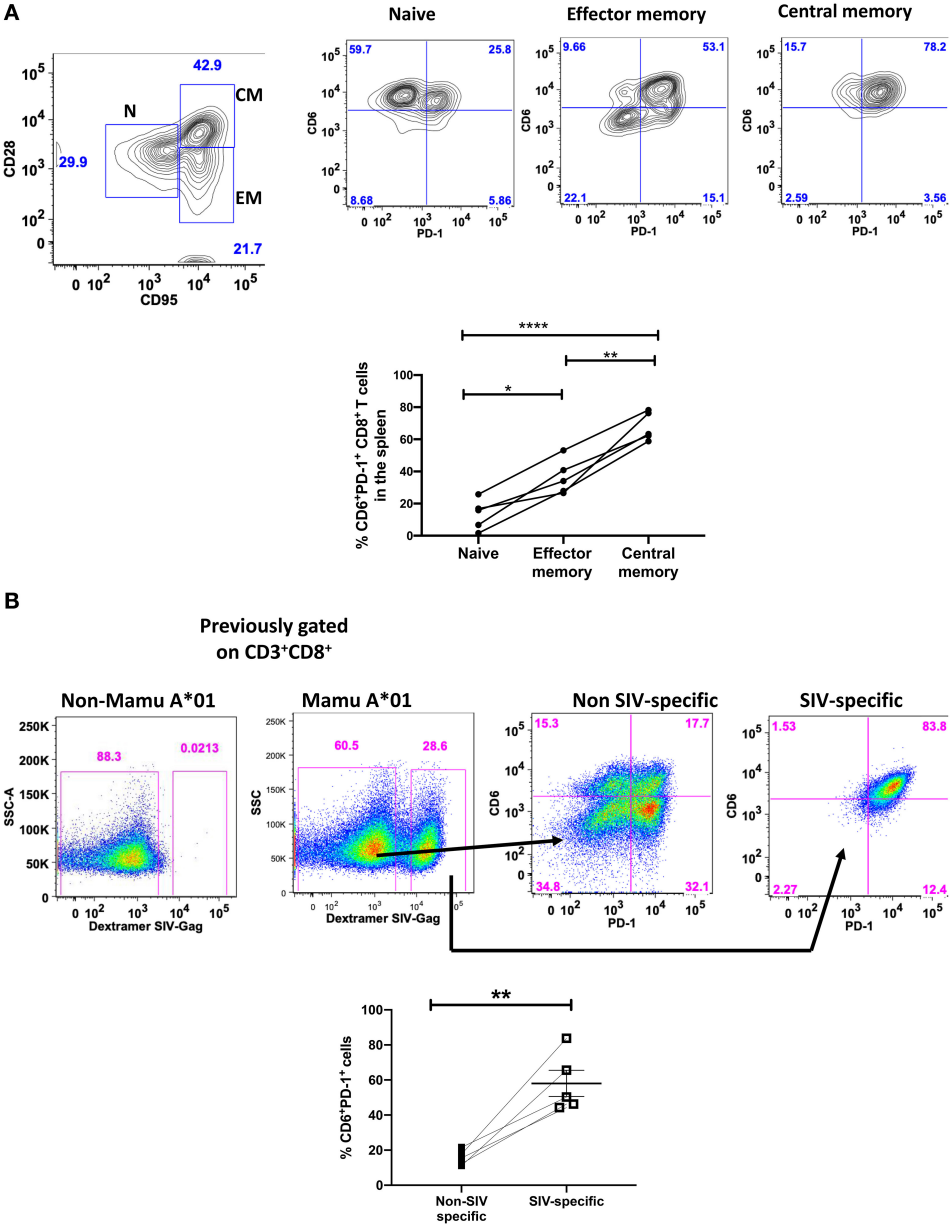
Co-expression of CD6 and PD-1 on memory and SIV-specific CD8^+^ T cells. **(A)** Flow cytometry gating strategy for CD6 and PD-1 co-expression on different splenic CD8^+^ T cell memory subsets. The frequency of CD6 and PD-1 co-expression on naïve, effector and central memory CD8^+^ T cells. Data are from 5 macaques. **(B)** Representative staining of CD6 and PD-1 co-expression on CM9-negative and CM9-specific splenic CD8^+^ T cells. Data are representative of 5 macaques. Shown at the bottom are the frequencies of CD6 and PD-1 co-expression within non-SIV specific and SIV-specific CD3^+^CD8^+^ splenic T cells. SIV-specific are defined as CD3^+^CD8^+^ CM9^+^, whereas non-SIV specific are defined as CD3^+^CD8^+^ CM9^−^. Data are shown as means ± SEM. For statistical analysis, Mann-Whitney tests were performed. *p < 0.05, **p < 0.01, ****p < 0.0001.

Central and effector memory CD8^+^ T cells are critically important in curtailing SIV disease progression following acquisition (34–36), therefore we assessed the co-expression of CD6 and PD-1 on different memory CD8^+^ T-cell subsets during the chronic phase of infection (Figure 3A). We found the greatest representation within the central memory population (CD28^+^CD95^−^), followed by effector memory (CD28^−^,CD95^+^), with the least expression in the naïve CD8^+^ T-cell population in SIV-infected macaques. Collectively these data showed that CD6 co-expresses with PD-1 preferentially on memory CD8^+^ T-cells compared to naïve CD8^+^ T-cells during chronic SIV infection.

Chronic viral infection often results in exhaustion of virus-specific CD8^+^ T-cells. SIV-specific CD8^+^ T-cells express high levels of PD-1 during the chronic phase of infection (37). We examined the expression of CD6 and PD-1 on SIV-specific CD8^+^ T-cells in the spleen identified by SIV Gag_181−−189_ CM9 dextramer staining of cells from Mamu A^*^01-positive macaques. We found the greatest representation of CD6 and PD-1 co-expression within the SIV-specific CD8^+^ T-cell compartment compared to limited expression within the non-SIV specific CD8^+^ T-cell compartment (Figure 3B). These results indicate that CD6 and PD-1 are also co-expressed on SIV-specific CD8^+^ T cells indicating that co-expression of CD6 and PD-1 may also identify dysfunctional virus-specific CD8^+^ T cells.

**Figure 4:**
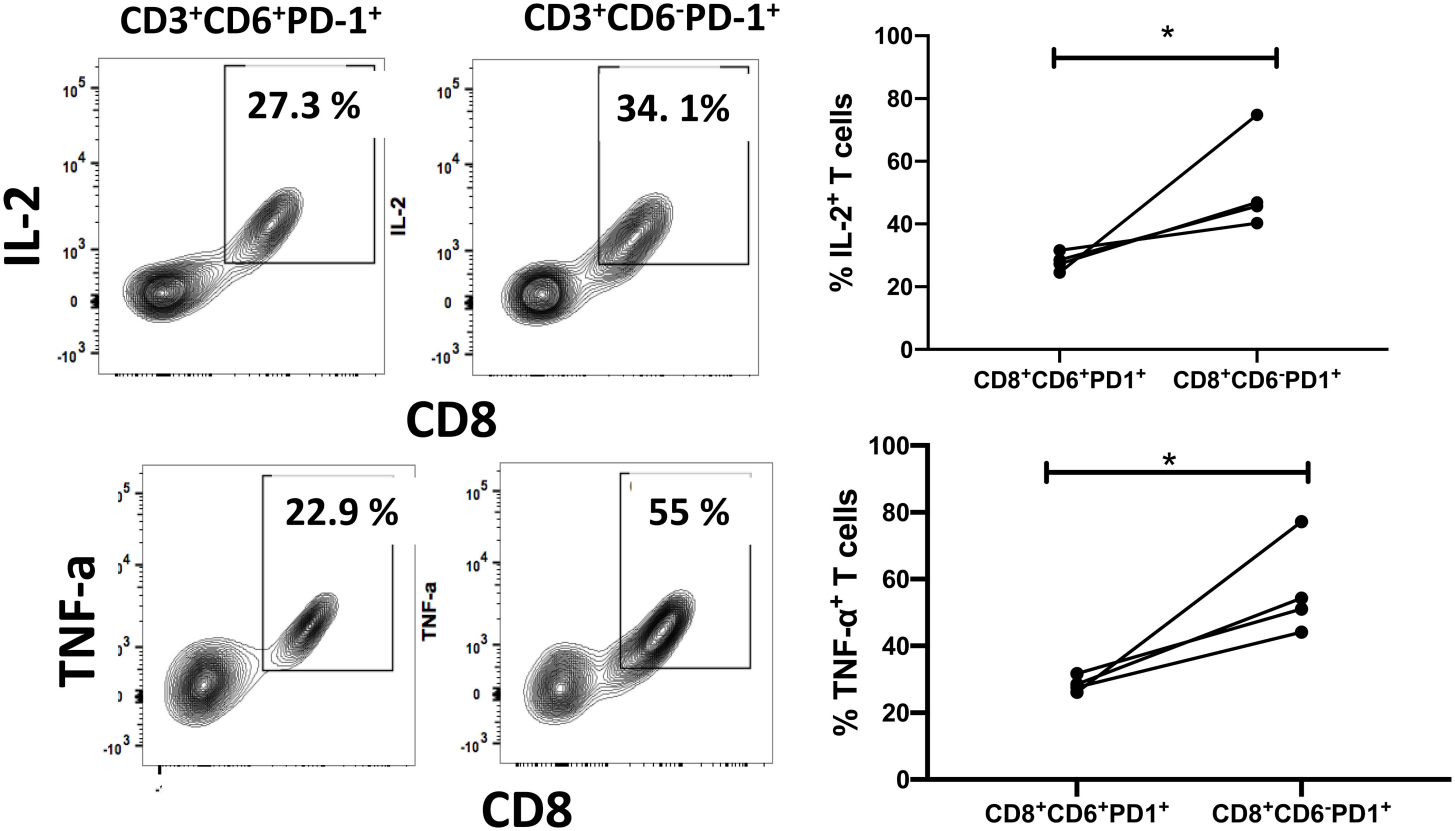
Overexpression of CD6 and PD-1 is associated with reduced cytokine secretion. Representative staining and the frequency of splenic CD6^+^PD-1^+^ and CD6^−^PD-1^+^CD8^+^ T-cells expressing IL-2 and TNF-α *in vivo*. Data are from 4 macaques. Data are shown as means ± SEM. For statistical analysis, Mann-Whitney tests were performed. *p < 0.05.

**Figure 5:**
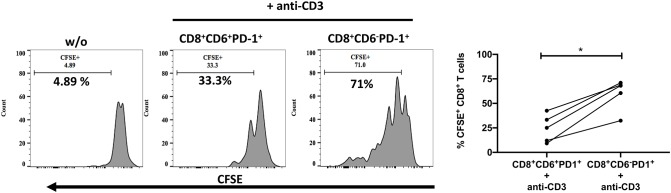
Overexpression of CD6 and PD-1 is associated with reduced proliferation. Representative staining and the frequency of splenic CD8^+^CD6^+^PD-1^+^ and CD8^+^CD6^−^PD-1^+^ T-cell CFSE dilution on day 3 following *in vitro* anti-CD3 stimulation. Data are from 5 macaques. Data are shown as means ± SEM. For statistical analysis, Mann-Whitney tests were performed. *p < 0.05.

**Figure 6:**
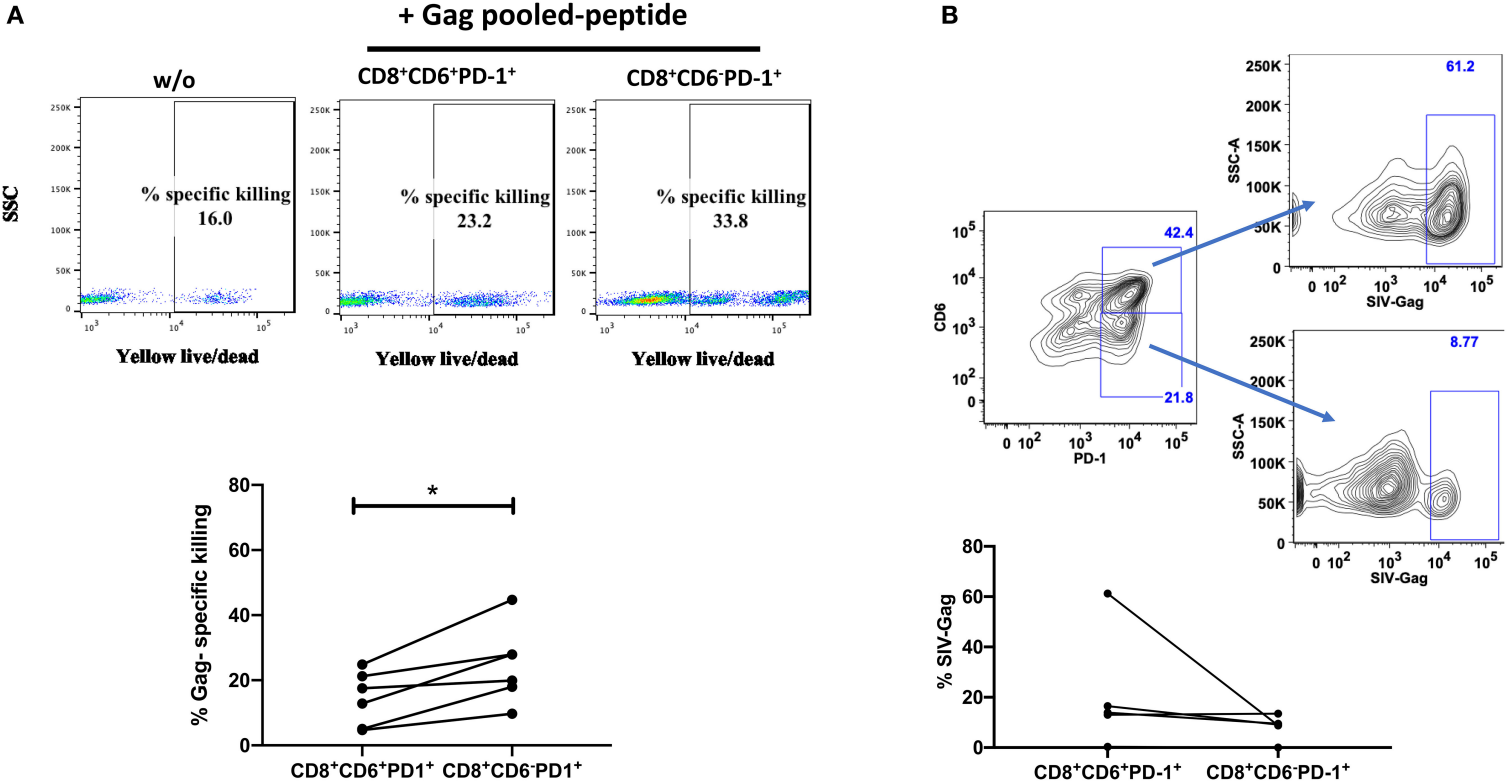
Overexpression of CD6 and PD-1 is associated with reduced cytotoxicity. **(A)** Representative staining of Gag-specific killing of autologous CD4^+^ T-cells by splenic CD8^+^CD6^+^PD-1^+^ and CD8^+^CD6^−^PD-1^+^ T-cells with and without pooled Gag peptides and the overall killing frequencies calculated as the peptide-stimulated response minus the unstimulated response. Data are from 6 macaques. **(B)** Representative staining and frequency of SIV-specific CD8^+^ T cells within the sorted splenic CD3^+^CD8^+^CD6^+^PD-1^+^ and CD3^+^CD8^+^CD6^−^PD-1^+^ T-cells. SIV-specific CD8^+^ T cells were detected by CM9 (Gag, CTPYDINQM; residues 181-189 FITC) dextramer staining. SIV-specific cells are defined as CM9^+^. Data are from 5 macaques. Data are shown as means ± SEM. For statistical analysis, Mann-Whitney tests were performed. *p < 0.05.

We next compared anti-viral functional properties of CD6^+^PD-1^+^ and CD6^−^PD-1^+^ CD8^+^ T-cells, including cytokine secretion, proliferation, and cytotoxic activity. IL-2, IFN-γ and TNF-α secretion is lost upon CD8^+^ T-cell exhaustion during chronic infection (38). We examined the intracellular cytokine levels of IL-2, TNF-α, and IFN-γ within CD6^+^PD-1^+^ and CD6^−^PD-1^+^ CD8^+^ splenocytes from chronically infected macaques *ex vivo* in the absence of any additional stimuli. The frequencies of IL-2^+^ and TNF-α^+^ cells were significantly diminished in the CD6^+^PD-1^+^ subset compared with the CD6^−^PD-1^+^ subset (Figure 4). Further, the proliferative capacity, determined by CFSE dilution following TCR crosslinking, of the CD6^+^PD-1^+^ subset was significantly reduced 2-fold compared to the CD6^−^PD-1^+^ subset following *in vitro* stimulation (Figure 5). Finally, sorted effector CD8^+^CD6^+^PD-1^+^ splenocytes from chronically infected macaques killed autologous CD4^+^ Gag peptide-pulsed target cells significantly less efficiently compared to CD8^+^CD6^−^PD-1^+^ splenocytes (Figure 6A).

**Figure 7:**
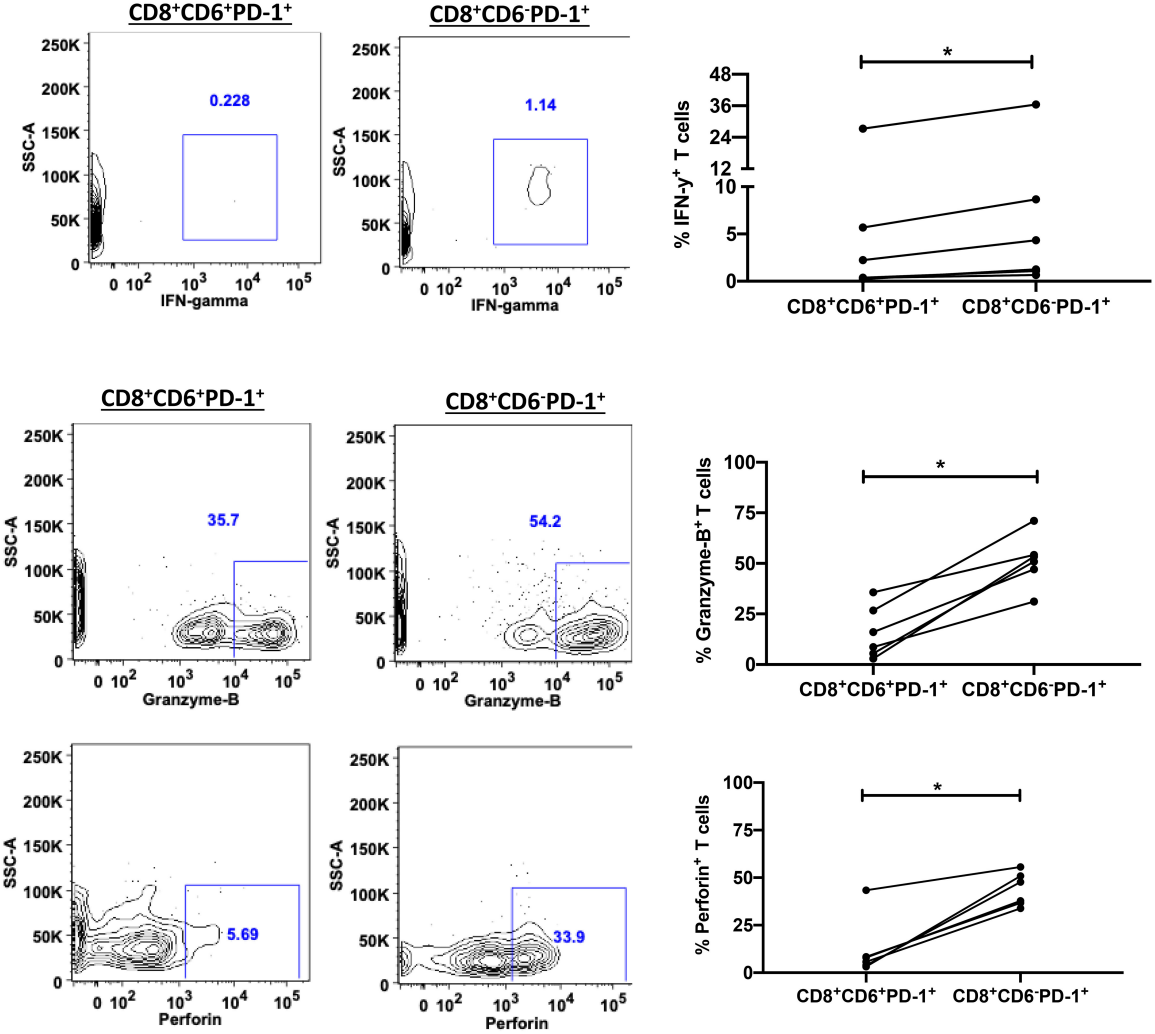
Overexpression of CD6 and PD-1 impairs CD8^+^ T cell antiviral activity. Representative staining and the frequency of splenic CD6^+^PD-1^+^ and CD6^−^PD-1^+^CD8^+^ T-cells expressing IFN-γ (Top), granzyme B (Middle) and perforin (bottom) *in vivo*. Data are from 6 macaques. Data are shown as means ± SEM. For statistical analysis, Mann-Whitney tests were performed. *p < 0.05.

The reduced cytotoxicity might have been due to a lower frequency of SIV-specific CD8^+^ T-cells within the sorted effector CD6^+^PD-1^+^CD8^+^ splenocytes. To exclude this possibility, we assessed the frequencies of SIV-specific CD8^+^ T-cells in CD8^+^ CD6^+^PD-1^+^ and CD8^+^ CD6^−^ PD-1^+^ T-cells. Interestingly, we found that the frequency of SIV-specific CD8^+^ T-cells was elevated in CD8^+^ CD6^+^PD-1^+^ cells however there was no significant difference between the two groups (Figure 6B). These data indicate that the differences in cytotoxicity were due to differences in functionality of these cells rather than differences in SIV-specific CD8^+^ T-cell frequencies. The secretion of IFN-γ, granzyme B and perforin play an essential role in cytotoxic CD8^+^ T cell-mediated killing of viral-infected cells. Therefore, we assessed the intracellular levels of INF-γ, granzyme B, and perforin between CD8^+^CD6^+^PD-1^+^ and CD8^+^CD6^−^PD-1^+^ T cells. We found that the frequencies of INF-γ^+^, granzyme B^+^, and perforin^+^ cells were significantly lower in the CD6^+^PD-1^+^ compared to the CD6^−^PD-1^+^ subset (Figure 7). Taken together, overexpression of CD6 and PD-1 severely compromised CD8^+^ T-cell anti-viral immune responses.

### CD6 Contribution to T-Cell Dysfunction May Be Dependent on SHP2 Pathway

**Figure 8:**
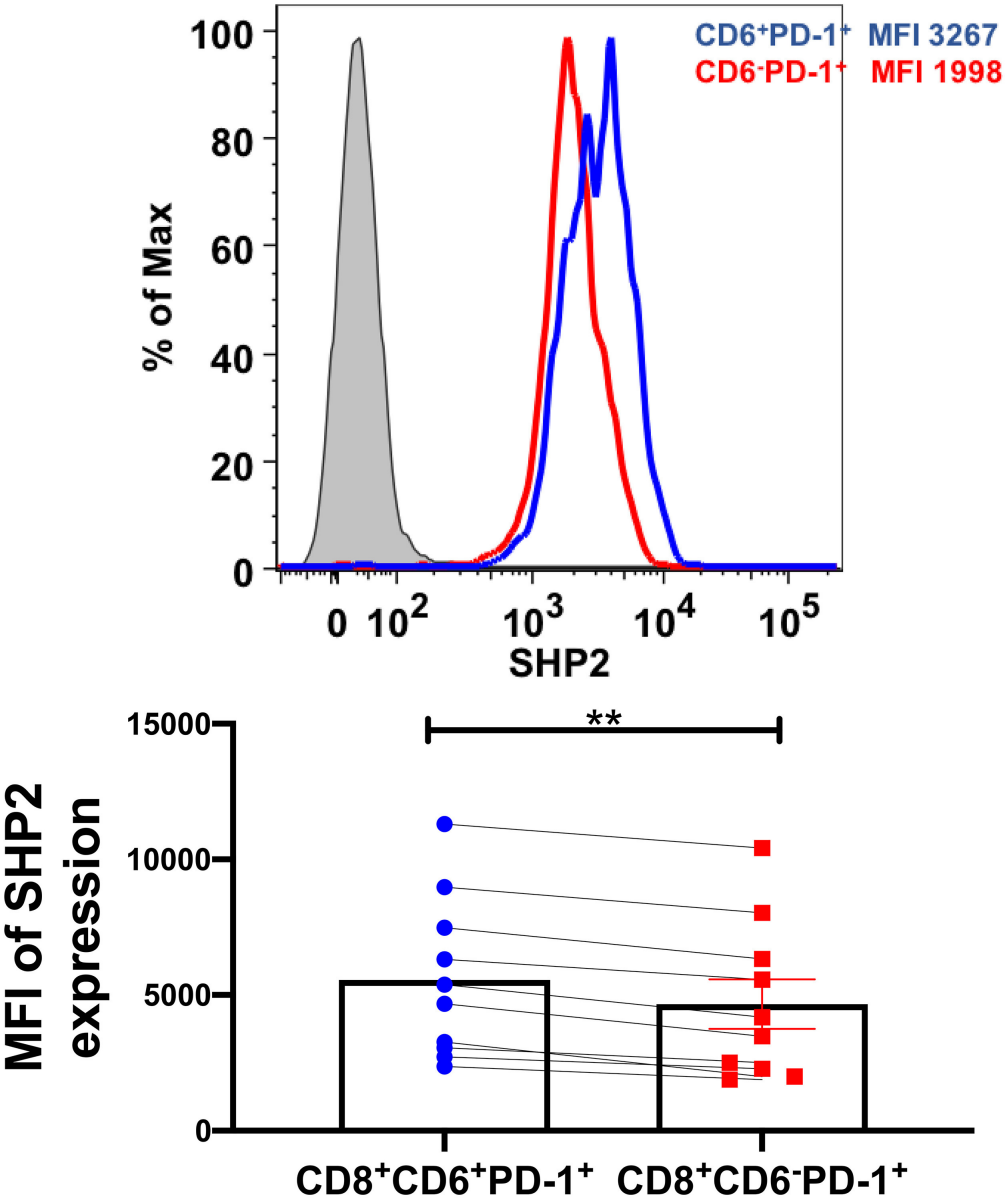
Expression of SHP2 in CD8^+^CD6^+^PD-1^+^ T-cells during chronic infection. Representative staining of SHP2 in CD8^+^CD6^+^PD-1^+^ and CD8^+^CD6^−^PD-1^+^ T-cells from chronically-infected macaques in the spleen. Median fluorescence intensity (MFI) of SHP2 in CD8^+^CD6^+^PD-1^+^ and CD8^+^CD6^−^PD-1^+^ T-cells (bottom panels). Data are from 10 macaques. Data are shown as means ± SEM. For statistical analysis, Mann-Whitney tests were performed. **p < 0.01.

CD6 interacts with the phosphatase SHP2 which plays a critical role in PD-1-mediated inhibition of T-cell activation. We investigated SHP2 expression and found significantly higher expression on CD6^+^PD-1^+^CD8^+^ T-cells (mean MFI of 5449) compared to CD6^−^PD-1^+^CD8^+^ T-cells (mean MFI of 4668) (Figure 8). These data suggest that CD6 may influence CD8^+^ T-cell dysfunction via a SHP2 dependent mechanism.

### CD6 and PD-1 Overexpression Is Associated With SIV Disease Progression

**Figure 9:**
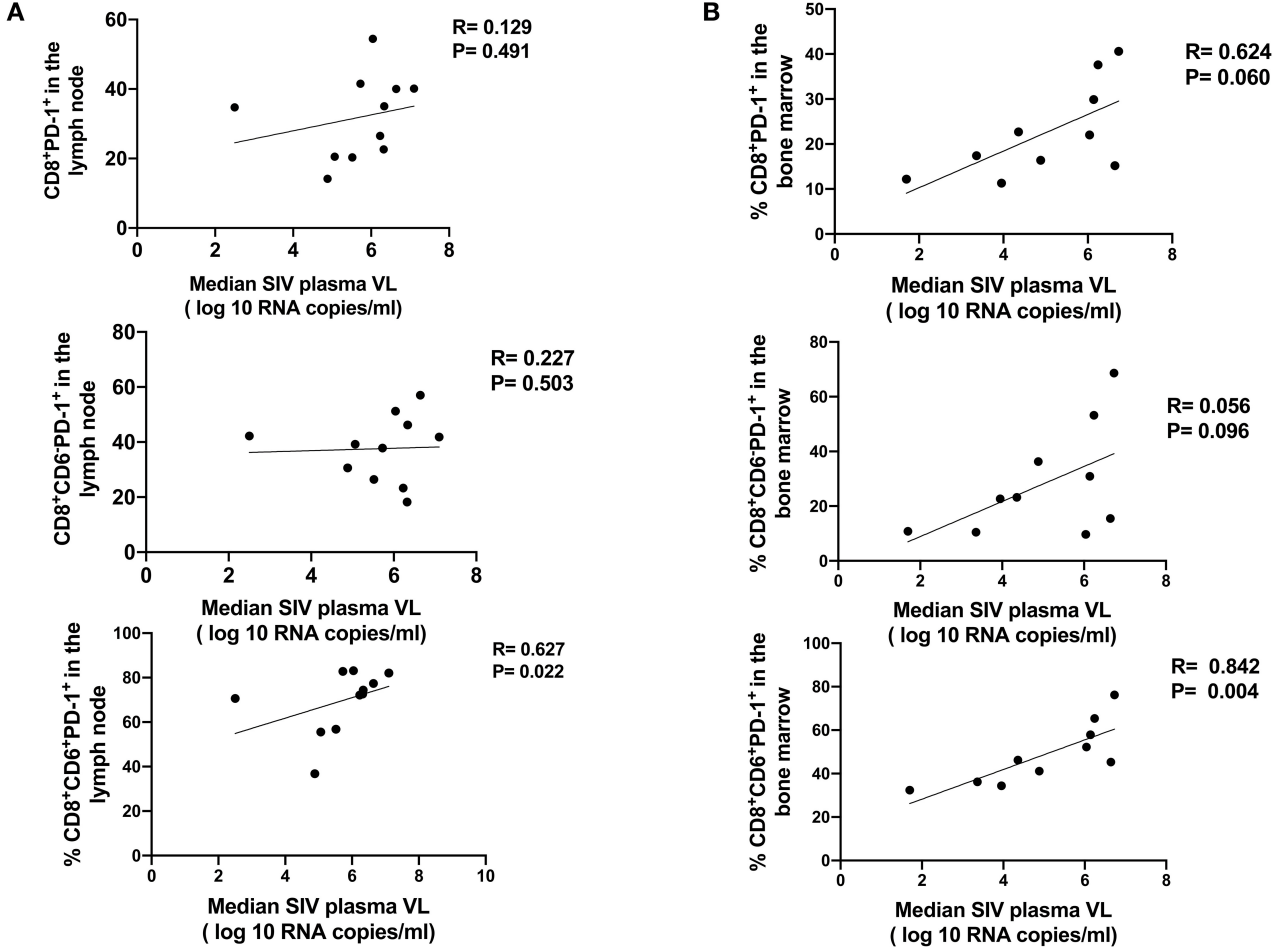
Overexpression of CD6 and PD-1 on CD8 T-cells is associated with SIV disease progression. The correlation of CD8^+^PD-1^+^ (Top), CD8^+^CD6^−^PD-1^+^ (Middle panel), CD8^+^CD6^+^PD-1^+^ (Bottom panel) T-cells in the **(A)** LN and **(B)** bone marrow with the median SIV plasma viral load (VL) over weeks 12–40 post-infection. Data are from 11 (lymph node) and 10 (bone marrow) macaques, respectively. For statistical analysis, the nonparametric spearman correlation was performed.

To determine whether the presence of CD6 on PD-1 expressing CD8^+^ T-cells during chronic infection contributes to disease progression, we assessed the relationship between the frequencies of various CD8^+^PD-1^+^ T-cell populations and SIV plasma viremia. Median viral loads were utilized because they provide a better representation of overall chronic viremia during disease progression and minimize random viral load fluctuations. No significant correlation between the frequency of either CD8^+^PD-1^+^ or CD8^+^CD6^−^PD-1^+^ T-cells in the LN and bone marrow and SIV median viral load was seen. However, a significant positive correlation between the frequency of CD8^+^CD6^+^PD-1^+^ T-cells in the LN and bone marrow and SIV median viral load was seen as anticipated (Figures 9A,B). As we found that SIV-specific CD8^+^ T cells highly express CD6 and PD-1, we assessed the relationship between the frequency of CD8^+^CD6^+^PD-1^+^ SIV^+^ T cells in the LN and plasma viral load. A positive correlation with viral load at the time of sample collection was observed but was not significant (Supplementary Figure 2), perhaps as only 4 Mamu A^*^01-positive macaques were available for analysis. An expanded study should investigate this association in depth in the future. Collectively, however, these data suggest that the presence of CD6 on CD8^+^PD-1^+^ T-cells is positively associated with SIV disease progression.

### *In vitro* CD6 and PD-1 Blockade Enhances CD8^+^ T Cell Proliferation

**Figure 10:**
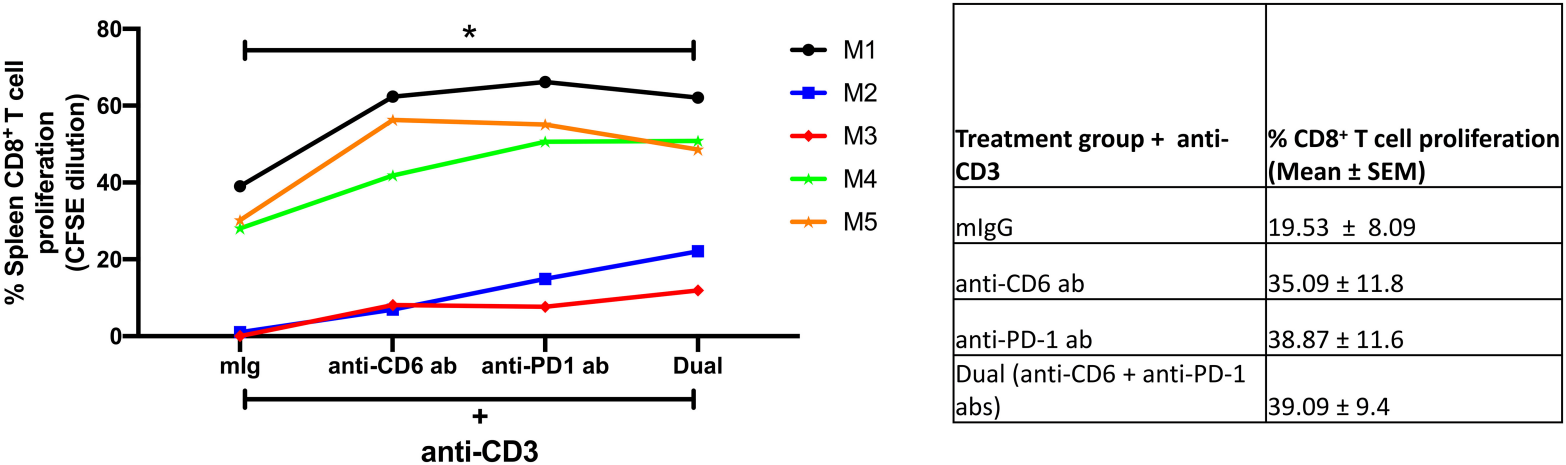
Effect of targeting CD6 and PD-1 on CD8^+^ T cell proliferation. The proliferation of chronic splenic CD8^+^ T cells on day 5 following *in vitro* soluble anti-CD3 stimulation in the presence of anti-CD6, anti-PD-1, anti-CD6 plus anti-PD-1(Dual), or mIg. Shown to the right are means ± SEM from 5 macaques. For statistical analysis, Kruskal-Wallis with Dunn's multiple comparison tests were performed. *p < 0.05.

To address whether blocking of CD6 and PD-1 may improve CD8^+^ T cell proliferation, we cultured splenocytes from chronically infected macaques with anti-CD3 for 5 days in the presence of anti-CD6 mAB (UMCD6), anti-PD-1 antibody, anti-PD-1 plus anti-CD6 antibodies, or control mouse Ig (mIg). The blocking of CD6 or PD-1 alone enhanced CD8^+^ T cell proliferation 1.8 and 2.0-fold, respectively, compared to control Ig (Figure 10). We also found that dual blocking of CD6 and PD-1 significantly enhanced CD8^+^ T cell proliferation 2.0-fold compared to control Ig (Figure 10). These data suggest that blocking CD6 and PD-1 can significantly increase the proliferative response of dysfunctional CD8^+^ T cells during chronic infection.

## Discussion

Here we show for the first time that CD6 and PD-1 co-expression defines a dysfunctional CD8^+^ T-cell population in the SIV rhesus macaque model. The co-expression of CD6 and PD-1 is abundant on memory and SIV-specific CD8^+^ T-cells. The frequency of CD8^+^ T-cells co-expressing CD6 and PD-1 was significantly expanded in peripheral lymphoid tissues of macaques in the chronic compared to acute phase of SIV infection. These CD6^+^PD-1^+^CD8^+^ T-cells displayed diminished proliferation, cytokine production, and cytotoxicity, highlighting their dysfunction. Their frequency positively correlated with SIV chronic viral loads suggesting a role in disease progression. Mechanistically, CD6^+^PD-1^+^CD8^+^ T-cells expressed elevated levels of SHP2 phosphatase providing a potential mechanism by which the presence of CD6 may restrict CD8^+^ T-cell anti-viral function during chronic SIV infection. Increased proliferation of splenocytes from chronically infected macaques in the presence of antibodies targeting both molecules suggested a potential therapeutic strategy for restoring CD8^+^ T cell function. These results identify a novel inhibitory pathway that potentially could be targeted *in vivo* for the reversal of T-cell dysfunction during SIV/HIV infection.

The T-cell CD6 expression profile in macaques was previously unknown. We found that the majority of splenic CD4^+^ T-cells expressed CD6 during chronic SIV infection, whereas CD6 expression separated CD8^+^ T-cells into two subsets, also observed in blood, bone marrow, LN, and BAL. Whether T-cells in mice and humans display similar expression profiles following viral infection is unknown. Although, PD-1 is rapidly upregulated on CD8^+^ T-cells *in vivo* following viral infection (39–41), studies on expression kinetics of CD6 on activated CD8^+^ T-cells *in vivo* are limited. Here, CD6 was quickly upregulated on LN CD8^+^ T-cells during acute infection and was sustained into the chronic phase of infection. In peripheral blood and bone marrow CD6 upregulation on CD8^+^ T-cells was restricted to chronic infection. Elevated expression of CD6 on CD8^+^ T-cells following viral infection is consistent with previous *in vitro* studies addressing the effect of TCR crosslinking on CD6 expression. Cell surface expression of CD6 on T-cells was increased after 48 h of stimulation and was sustained until late into Th17 polarization (19). Overall, our data provide novel insights into the *in vivo* dynamics of CD6 cell surface expression on CD8^+^ T-cells in different tissue compartments following viral infection.

Co-expression of PD-1 and inhibitory molecules such as TIM-3 (42) and LAG-3 (43) are associated with T-cell dysfunction during viral infection. Here, PD-1 predominantly co-expressed with CD6 during chronic infection, suggesting that CD6^+^PD-1^+^CD8^+^ T-cells might also express other inhibitory receptors. Importantly, expansion of CD8^+^CD6^+^PD-1^+^ T-cells in the LN, bone marrow, and BAL was restricted to chronic infection, suggesting that overexpression of CD6 and PD-1 on CD8^+^ T-cells might identify a subset of dysfunctional CD8^+^ T-cells that arises only during this phase. PD-1 over-expression has been associated with diminished T-cell effector function during chronic infection (40, 42–45). Our results showing that CD8^+^CD6^+^PD-1^+^ T-cells displayed impaired cytokine secretion, proliferation and cytotoxic capabilities compared to CD8^+^CD6^−^PD-1^+^ T-cells suggested a negative effect of CD6 expression on T-cell effector function consistent with a previous report in which T-cell proliferative responses and calcium influx were significantly elevated in CD6-deficient mice compared to wild-type controls (23). Additionally, over-expression of CD6 on human T-cells significantly impaired IL-2 secretion, calcium influx and proliferation following T-cell activation (24).

The inhibitory effect of CD6 has been attributed to its cytoplasmic domain, which lacks intrinsic catalytic activity but contains binding motifs for association with signaling molecules (24). CD6 interacts with SHP2 phosphatase (19, 46), central to PD-1 mediated suppression of T-cell responses following PD-1 ligand binding (25, 47). Moreover, SHP2 modulates select T-cell exhaustion features in chronic LCMV infection (48). Here, expression of SHP2 was significantly elevated on CD6^+^PD-1^+^CD8^+^ T-cells compared to CD6^−^PD-1^+^CD8^+^ T-cells, suggesting that CD6 over-expression on CD8^+^ T-cells during chronic infection may lead to formation of an inhibitory signaling hub composed of CD6, PD-1 and SHP2, resulting in attenuation of T-cell responses. This is an area for further investigation.

Defective anti-viral CD8 T-cell responses are associated with SIV/HIV disease progression (49). Here, the frequency of CD8^+^CD6^+^PD-1^+^ T-cells in the LN and bone marrow during chronic infection positively correlated with SIV viral load, consistent with their impaired cytokine production and proliferative responses, and also consistent with previous positive correlations between splenic CD8^+^CD6^+^PD-1^+^ T-cells and SIV plasma viral load and splenic B1 cells during chronic infection (50). The frequency of CD8^+^PD1^+^ or CD8^+^CD6^−^PD1^+^ T cells in the LN and bone marrow did not correlate with SIV viral load highlighting the important contribution of CD6 to the positive association with viremia.

CD6 is a therapeutic target for the treatment of several human autoimmune diseases (22). To address the therapeutic potential of targeting CD6 and PD-1 as an approach to reverse CD8 T cell dysfunction during chronic infection, we assessed the effect of blocking PD-1 and CD6 on splenic CD8^+^ T cell proliferation *in vitro*. Using splenocytes from chronically infected macaques, we found that dual blockade of CD6 and PD-1 significantly enhanced CD8^+^ T cell proliferation compared to control mIg blocking antibodies (Figure 10). This promising *in vitro* result should be substantiated in a larger cohort of SIV-infected macaques. Further, similar dual treatment should also be explored in other chronic immune diseases and malignant cancers where CD8^+^ T cell dysfunction/exhaustion plays a role in disease maintenance and progression.

In summary, the overexpression of CD6 and PD-1 on CD8^+^ T cells during the chronic phase of SIV infection marks dysfunctional anti-viral T cells. Preliminary studies suggest that the dual blockade of CD6 and PD-1 pathways may reverse CD8^+^ T cell dysfunction. Future mechanistic studies exploring the diminished functionality of CD6^+^PD-1^+^ CD8^+^ T cells should provide further avenues for improved treatment of individuals with HIV infection and other diseases displaying similar CD8^+^ T cell dysfunction.

## Data Availability Statement

All datasets generated for this study are included in the article/Supplementary Material.

## Ethics Statement

The animal studies were reviewed and approved by the Animal Care and Use Committees of Advanced BioScience Laboratories, Inc., and the National Cancer Institute.

## Author Contributions

GE-A and MR-G developed the concept and designed the experimental plans and prepared the manuscript. GE-A, AN, MR, and CH performed experiments. GE-A, AN, MR, SH, E-JK, RH, and TH processed tissue samples and provided data critique. GE-A and AN analyzed data. All authors reviewed the manuscript.

### Conflict of Interest

The authors declare that the research was conducted in the absence of any commercial or financial relationships that could be construed as a potential conflict of interest.

## Footnotes

^1^Musich T, Thovarai V, Fourati S, Venzon DJ, Mohanram V, Tuero I, Miller-Novak LK, Helmold Hait S, Rahman MA, Hunegnaw R, Huiting E, Yuan W, O'hUigin C, Hoang T, Cartwright M, Cameron MJ, Schifanella S, Doster MN, Vaccari M, Sui Y, Labranche C, Montefiori D, Bear J, Rosati M, Berzofsky JA, Pavlakis GN, Felber BK, Franchini G, Robert-Guroff M. In preparation.

^2^Helmold Hait S, Hogge CJ, Rahman MA, Ko E-J, Hunegnaw R, Mushtaq Z, Enyindah-Asonye G, Hoang T, Miller Jenkins LM, Appella E, Appella DH, Robert-Guroff M. A SAMT-247 microbicide provides potent protection against intravaginal SIV infection of rhesus macaques while an added vaccine component elicits mixed outcomes. Submitted for publication.
