# Supplementary material for: Overexpression of CD6 and PD-1 Identifies Dysfunctional CD8+ T-Cells During Chronic SIV Infection of Rhesus Macaques
Source: Front Immunol. 2020 Jan 8;10:3005. doi: 10.3389/fimmu.2019.03005 (PMC6961594; doi:10.3389/fimmu.2019.03005)
Supplement: Supplementary file 1 [file Data_Sheet_1.pdf]

Supplementary Figure 1

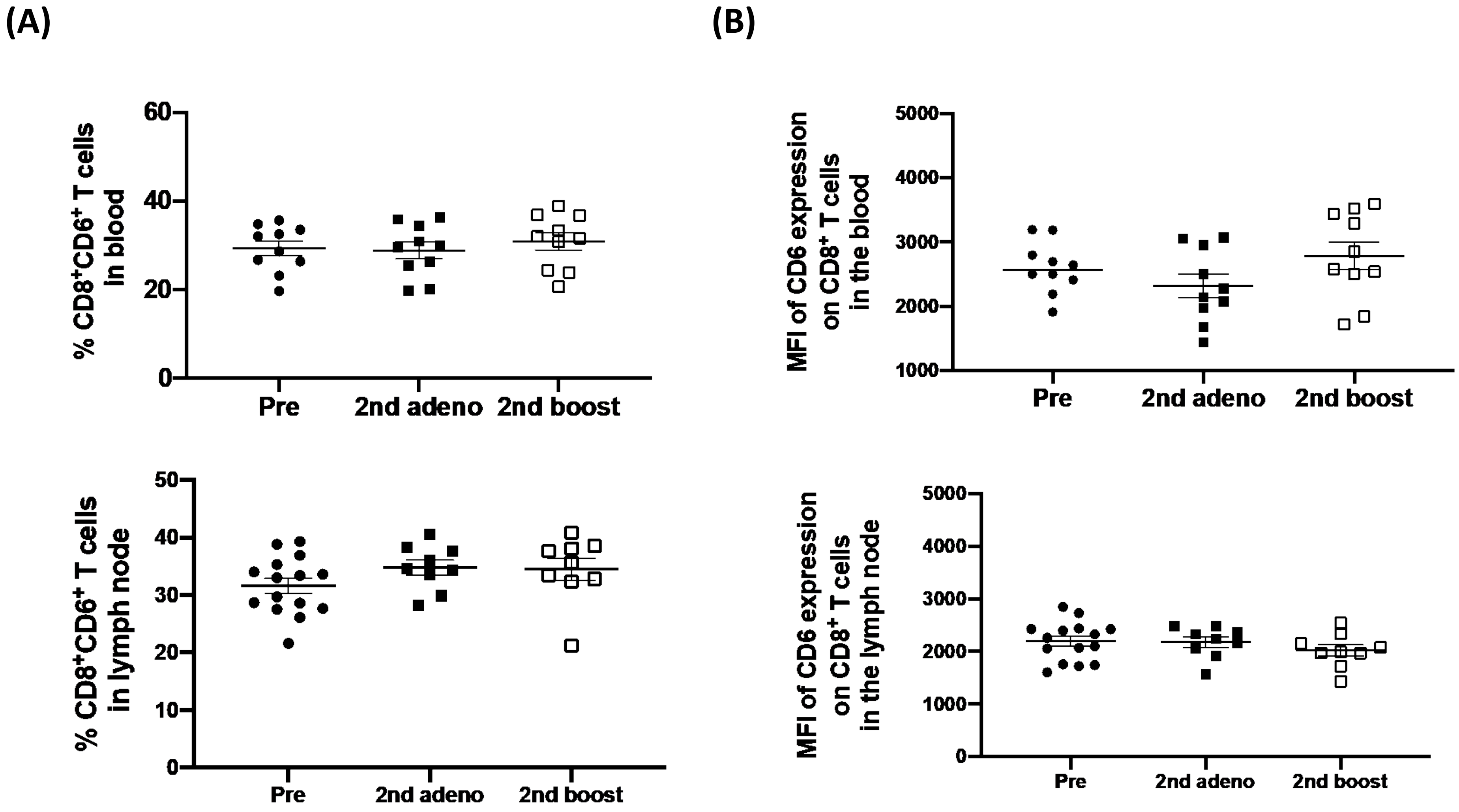

Supplementary Figure 1. Dynamics of CD8^{+}CD6^{+} T cells during vaccination. (A) The frequency of CD8^{+}CD6^{+} T cells in the blood (Top) and lymph node (bottom) at different time points following vaccination. (B) The expression levels of CD6 on CD8^{+} T cells in the blood (Top) and lymph node (bottom) at different time points following vaccination. 2nd adeno (at week 14; 2 weeks post 2nd Adeno immunization), 2nd boost (at week 38; 2 weeks post 2nd protein boost).

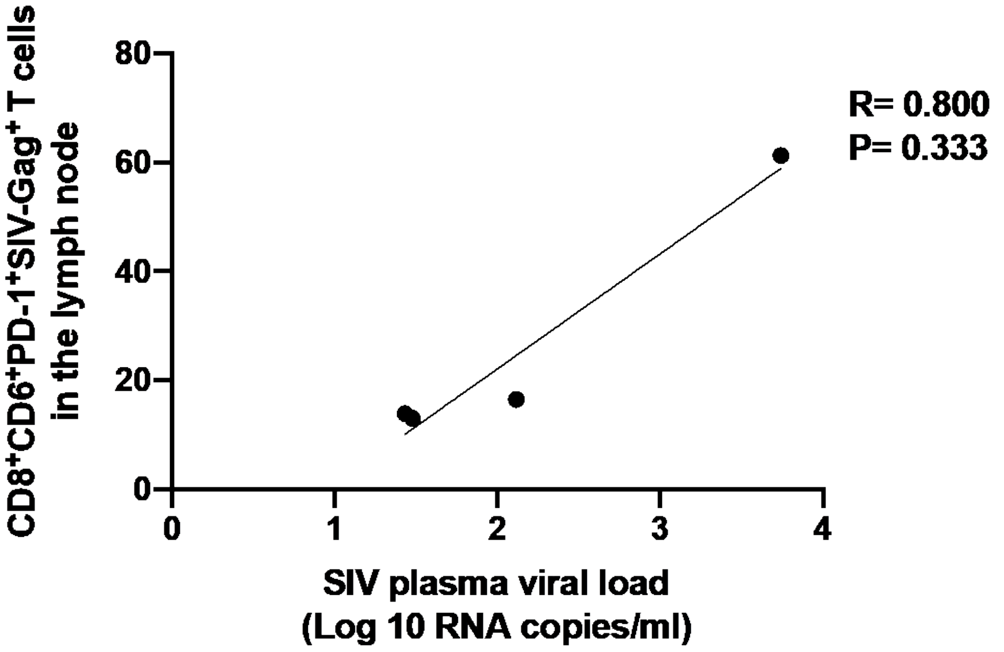

Supplementary Figure 2. Correlation of SIV-specific $CD8^+$ T cells expressing CD6 and PD-1 with plasma viral load. The correlation of $CD8^+CD6^+PD\text{-}1^+$ SIV^+ T cells in the lymph node with the SIV plasma viral load at the time of sample collection (~ 40 weeks post-SIV infection). Data are from 4 macaques. For statistical analysis, the nonparametric spearman correlation was performed.
